# Genomic selection needs to be carefully assessed to meet specific requirements in livestock breeding programs

**DOI:** 10.3389/fgene.2015.00049

**Published:** 2015-02-20

**Authors:** Elisabeth Jonas, Dirk-Jan de Koning

**Affiliations:** Department of Animal Breeding and Genetics, Swedish University of Agricultural Sciences, Uppsala, Sweden

**Keywords:** breeding, estimated breeding value, marker-assisted selection, generation interval, modeling, non-additive effects

## Abstract

Genomic selection is a promising development in agriculture, aiming improved production by exploiting molecular genetic markers to design novel breeding programs and to develop new markers-based models for genetic evaluation. It opens opportunities for research, as novel algorithms and lab methodologies are developed. Genomic selection can be applied in many breeds and species. Further research on the implementation of genomic selection (GS) in breeding programs is highly desirable not only for the common good, but also the private sector (breeding companies). It has been projected that this approach will improve selection routines, especially in species with long reproduction cycles, late or sex-limited or expensive trait recording and for complex traits. The task of integrating GS into existing breeding programs is, however, not straightforward. Despite successful integration into breeding programs for dairy cattle, it has yet to be shown how much emphasis can be given to the genomic information and how much additional phenotypic information is needed from new selection candidates. Genomic selection is already part of future planning in many breeding companies of pigs and beef cattle among others, but further research is needed to fully estimate how effective the use of genomic information will be for the prediction of the performance of future breeding stock. Genomic prediction of production in crossbreeding and across-breed schemes, costs and choice of individuals for genotyping are reasons for a reluctance to fully rely on genomic information for selection decisions. Breeding objectives are highly dependent on the industry and the additional gain when using genomic information has to be considered carefully. This review synthesizes some of the suggested approaches in selected livestock species including cattle, pig, chicken, and fish. It outlines tasks to help understanding possible consequences when applying genomic information in breeding scenarios.

## INTRODUCTION

Plant and livestock breeding started many 1000 years ago with the cultivation of plants and capture of individual animals, leading to the domestication of most agricultural species used today. Early approaches were based on phenotypic evaluations with little awareness of the underlying causes of different productivities and appearances. This was changed substantially when more information on the genetic background became available, firstly without and later by using genetic marker information. Basic methods were developed after the (re-)discovery of the Mendelian laws of inheritance, and further advances were based on the knowledge on inheritance of genetic information via linkage and linkage disequilibrium. Theoretical approaches and progression on the field of quantitative genetics, especially the work from Fisher, Wright and Lush, allowed multifactorial models and inclusion of complex pedigrees into defined breeding decisions (for example reviewed in Hill and Mackay, 2004; Hill, 2014). A combination of theoretical approaches and experimental achievements has played a significant role for the development of modern breeds. Today, structured breeding programs exist for most livestock species. They rely on the routine recording of pedigree and performance information on populations and they further implement the basic knowledge on inheritance into selection choices for the progression of the selected population. Breeding programs for different species and production systems apply different strategies and are built in different ways. Many livestock breeding populations (e.g., chicken, fish, pig, beef cattle) are structured in tiers of nucleus, multiplier and commercial farms, which are commonly shaped as a pyramid with few nucleus breeders and many commercial breeders (Nicholas, 2009). Genetic progress does mainly occur in the upper tier, and improved breeding stock is transferred by relatively few nucleus breeders to the multipliers. The flow of genes is directed downward and commercial farmers rely on both improvement in the nucleus herds as well as little loss of genetic progress during the multiplication of animals (Nicholas, 2009). Open-nucleus schemes have the advantage of a lower inbreeding rate and potentially increased selection response compared to a closed-nucleus. In a closed nucleus, the breeding company maintains the full ownership of the elite breeding material.

**FIGURE 1:**
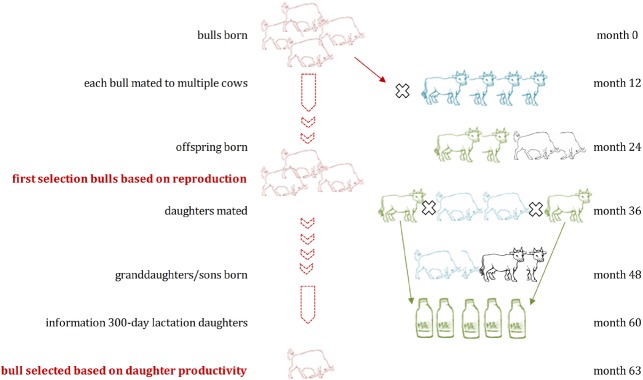
**Schematic overview of traditional breeding in dairy cattle: Selection candidates (bulls) are born and mated to multiple cows at around 12 months of age.** In month 60 the first phenotypic recordings (milk yields) are available from the daughters of the selection candidate, as they had to reach puberty, be mated and finished a first lactation. Estimated breeding values are available after approximately 63 months and the bulls for further breeding are selected.

**FIGURE 2:**
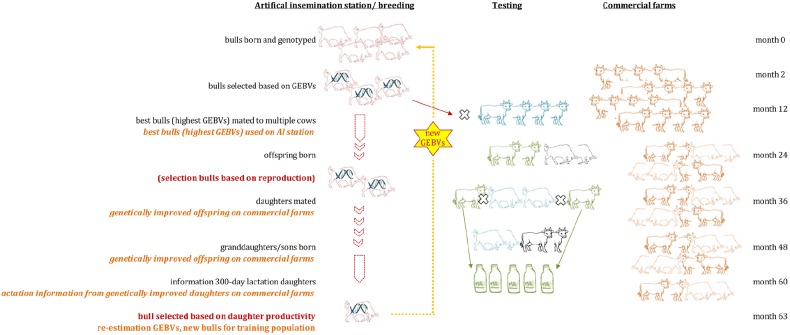
**Marker enhanced breeding using Genomic Selection in dairy cattle: Genome-wide marker panels are used to identify association or linkage with traits of interest using bulls with information on daughter lactation and genotypic data.** Estimated effects of each marker are summarized into genomic estimated breeding values (GEBV), which can be used for selection. The figure assumes that GEBVs are available from an existing training population in month 0 and are being updated in month 63 with additional information. Only selected animals (based on GEBVs) are used for testing. Semen of superior bulls can be used earlier on commercial farms, allowing for higher genetic gain. Genomic selection allows a significant reduction of the generation interval from 6 to 1.5 years if applied as suggested in the figure.

Livestock breeding programs use estimated breeding values (EBVs), mainly estimated for sires, for selection decisions based on own performance and that of relatives. EBVs are estimated using traits measured in an own-performance, pedigree, sib, and/or progeny evaluation scheme in specialized testing stations and/or selected farm environments using, most of the times, best linear unbiased predictions (BLUP; Henderson, 1975). The breeder’s equation is an indicator for the change due to selection (Δ*Z* = *h*^2^ × S, where Δ*Z* is the change of the mean of a quantitative trait in one generation, heritability *h*^2^ describing the phenotypic similarity between relatives or trait variation due to additive genetic effects, and S measures the selection intensity). It indicates that the change of a quantitative trait depends on its heritability and selection intensity (Falconer and Mackay, 1996; Lynch and Walsh, 1998; Xu and Hu, 2010). The evaluation of the genetic merit targets the sires in many livestock species (i.e., in cattle or pigs). Sires have more offspring, especially when artificial insemination (AI) is used, and their genetic impact on livestock populations is therefore stronger (Gerrits et al., 2005; Funk, 2006). AI stations and/or nucleus breeders play therefore the main role for improvement of the genetic merit in a population. Generation intervals of 4 to 5 years in beef cattle, more than 5 years in dairy cattle (Figure 1) and 2 to 2.5 years in pig breeding schemes are one consequence of the time-consuming evaluation of phenotypes to enable a reliable estimation of breeding values (Schefers and Weigel, 2012). The advances of having sophisticated breeding programs implemented, are diminished by the long time period until a new generation is available for breeding (often defined as the average age of selected parents when the offspring is born or generation interval), and costs for trait recording.

## BREEDING USING MOLECULAR GENETIC MARKERS

The goal of most breeding programs is to predict the genetic merit of an individual and thus allow targeted combinations of desired alleles to improve the performance of the next generation(s). The phenotype of an individual is only in rare cases a good indicator for allelic differences, while the use of genetic markers allows tracing detailed information on the inherited part of the genome, other than such observed by the phenotype (Botstein et al., 1980). Today, genetic DNA markers are used to assist breeding and selection and to build the basis for novel breeding approaches such as marker-assisted selection (MAS) and more recently GS (Williams, 2005). MAS, which uses one or few markers as a selection tool, has not reached the initial proposed achievements, the main reason is that the discovery of reliable markers is difficult, especially when working with complex traits. Only relatively few consistent markers could be identified, despite successful identification of many quantitative trait loci (QTL; Hu and Reecy, 2007). Advancements of molecular genetic tools, allowing genotyping of samples at many loci at a time, further advanced the aim of using genetic markers for selection. Meuwissen et al. (2001) suggested GS, which is based on the use of genome-wide markers panels to derive breeding values based on genomic information. Genomic EBVs (GEBVs) or predictions of the genetic merit of an individual based on its genome are derived for candidates with genotype and phenotype information, the so-called training population. The information is then used for the selection of genotyped candidates with no recorded traits (selection candidates). GS can be applied in practice for all main livestock species since genome-wide single nucleotide polymorphism (SNP) panels or even full sequence information are available (Table 1). GS has been incredibly successful in dairy cattle, where GEBVs are published in a number of countries^1^. Different prediction methods (linear models such as BayesA, BayesB, GBLUP etc., non-linear models such as Neural Networks or Reproducing Kernel Hilbert spaces; penalized methods including ridge regression or Bayesian shrinkage estimation) applied to a wide range of datasets have been reviewed previously and suggestions of implementations were discussed and can be found elsewhere (e.g., Daetwyler et al., 2012; de los Campos et al., 2012; Morota and Gianola, 2014). However, beside general prediction methods also some hurdles to be taken before this selection approach can be of practical use in livestock populations other than dairy cattle have been described (Ibanez-Escriche and Gonzalez-Recio, 2011). Especially the basic breeding programs, accuracies of EBVs and possibilities and needs of implementing non-additive effects into genomic predictions differ between populations. This might also have an effect on marker density required and thus genotyping costs, additionally might the value of each individual selection candidate be relatively low, compared to an elite breeding bull (Ibanez-Escriche and Gonzalez-Recio, 2011).

**TABLE 1:** **Overview of genome structure and genotyping platforms of the main livestock species**.

**Species**	**Ploidy**	**Genome size [Mb]**	**Number of genes**	**Genome structure**	**Linkage disequilibrium**	**Commercial arrays**	**Reference sequence**
Cattle (*Bos taurus*)	Diploid (2n = 60)	∼2870	∼26835	3000000 SNP identified	Highly variable extent of LD	Illumina: 54609 SNP, Affymetrix: 640000 SNP	Hereford cow (whole genome shotgun), her sire (hierarchical BAC clone; Elsik et al., 2009)
Pig (*Sus scrofa*)	Diploid (2n = 38)	∼2596	∼21640	510000 SNP identified	Higher LD (than some Holstein cattle)	Illumina: 64232 SNP	Female domestic Duroc pig (Illumina whole-genome shotgun, BAC clone; Groenen et al., 2012)
Chicken (*Gallus gallus*)	Diploid (2n = 78)	∼1000	20000–23000	1800000 SNP identified	Difference of LD between layer lines	Affymetrix: 580000 SNP	Single red jungle fowl female from inbred line; ∼6.6 × whole-genome, shotgun reads BAC-end read pairs (Int Chicken Genome Sequencing, 2004)
Atlantic salmon (*Salmo salar*)	Diploid (2n = 58)	∼6000	33709 (identified in 2010)	Many chromosomal rearrangements	Moderate LD	iSelect Atlantic salmon 16,500 SNP	Female fish; aimed end of 2013 (Davidson et al., 2010); announced finish June 2014

SNP, single nucleotide polymorphism; LD, linkage disequilibrium; BAC, bacterial artificial chromosome.

### APPLICATION OF GENOMIC SELECTION IN DAIRY CATTLE

Dairy cattle are mostly bred based on a rather simple within-breed selection principle. Occasionally crossbred calves are produced which can then be used for beef production if no replacement heifers are needed. Selection is mainly performed on the sire-side; bulls have a much greater contribution to the national and international breeding stock as their semen is distributed via AI. Despite the success of breeding programs in improving traits such as milk yield, limitations of traditional selection have been suggested. Increased health and fertility problems, the relative importance of dairy cattle production world-wide, the long generation interval due to the long reproductive cycle and the indirect measurement of the main traits of interest via the daughters of a bull made dairy cattle a good model for the implementation of GS. Further early efforts to design genotyping panels and to assemble the full genome sequence (Table 1), as well as the relatively closely linked world-wide population of the main breed, Holstein-Friesian, were reasons that GS was firstly employed in dairy cattle. An additional benefit was that GS could be implemented within the existing breeding and recording structures. Costs and generation intervals of traditional breeding using progeny testing are considerable and the improvements provided when using GS were prospected to have an incredible impact on the dairy industry in the future (Hayes et al., 2009; Thomasen, 2013).

**FIGURE 3:**
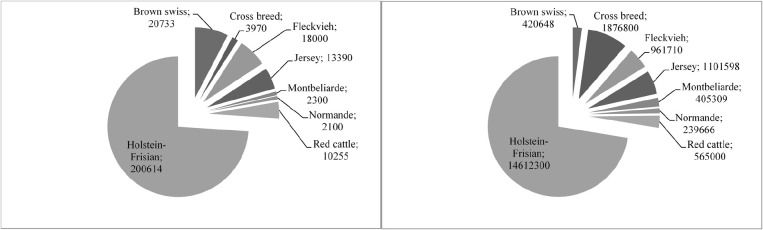
**Numbers of animals in active genomic selection breeding programs adapted from** (Thomasen, 2013). Shown is the total number of bulls in the reference panel **(A)** and recorded cows **(B)**.

Generation intervals were suggested to drop from around 5 to 6 years in traditional dairy cattle breeding programs to around 1.5 years (Figure 2) when using GS (Pryce and Daetwyler, 2012). It has been predicted that the costs for testing of bulls can be drastically reduced as GEBVs are available at birth, leading to an improved genetic change among other possible advances (Schaeffer, 2006). Costs for genotyping and thus the implementation of GS in dairy cattle is also cheaper since mainly male animals are being genotyped and semen of bulls is often distributed across multiple countries. Reasons for that are that AI is commonly used in dairy cattle populations, semen storage over a long time is possible in cryopreservation, and preferred bulls have therefore a higher impact on the breeding stock. Many breeding programs focus on the selection of sires while using phenotypic information from cows only for the derivation of EBVs (and GEBVs) for bulls. Only a few breeding programs include cows into their reference population to better assess additional traits and increase the reference population (Figure 3).

Genomic selection has been introduced into dairy cattle breeding programs rapidly and GEBVs are published in a number of countries (see text footnote 1). The integration of GS into breeding schemes has been reviewed recently (Pryce and Daetwyler, 2012; Bouquet and Juga, 2013). There are, in general, two options for the integration of genome-wide selection into breeding decisions, by either pre-selecting young bulls for further testing (pre-selection scheme) or for selection of new breeding bulls based on genomic information only (turbo scheme). However, especially due to the reduced generation interval (as in the “turbo” scheme), genomic inbreeding rates need to be observed and managed. The pre-selection scheme offers a rather conservative method with no change to the breeding program while producing moderate genetic gain, while the turbo scheme allows high genetic gain by considerably changing the structure of the breeding program (Bouquet and Juga, 2013). Details of the design of commercial breeding schemes with implemented GS are mostly unknown as most breeding companies might keep and test their strategy first in detail as this methodology has yet to be fully established (Pryce and Daetwyler, 2012).

### APPROACHES IN BEEF CATTLE

In beef cattle breeding, selection indices are often based on a specified market. Economic weights given to each selection target assist to combine the EBVs for different traits for the selection of the breeding stock. EBVs are, depending on the trait and number of information used, relatively good predictors for the future productivity of breeding stock. However, current breeding values are still less accurate compared to those in dairy cattle (Johnston et al., 2012). Genetic markers are commercially licensed for some traits in beef cattle, but many markers used for trait improvement do not perform consistently across populations (Allan and Smith, 2008). The use of genome-wide marker sets has therefore been of major interest for beef cattle breeding. Traditional breeding using progeny testing focusses largely on bulls at the nucleus level. An extended inclusion of cow information into the selection process would allow farmers to select superior dams (Saatchi et al., 2012) and lead to a breeding program which allows a more balanced inclusion of all traits considered in the breeding goal (Garrick, 2011). However using GS to select stock, especially cows, on the farm level is often restricted to purebred operations and pre-selection of cows would still be required to keep costs at a reasonable level. Only a cost-efficient strategy, for example by providing high accuracy of prediction, will allow successful use of GS also in beef cattle populations (Johnston et al., 2012). Heritability of the economically most important traits, training population (or total number of bulls with reliable EBVs) as well as the effective population size are factors influencing the applicability of genetic markers in beef cattle and determine also the great differences to the currently observed successes in dairy cattle (Johnston et al., 2012).

Overcoming especially the problem of small training populations would require a combination of data across countries and/or across breeds, and thus, higher density marker panels would be required to allow reliable predictions (de Roos et al., 2009). It has been hypothesized that a higher marker density, which would allow the inclusion of causative mutations or markers perfectly inherited with such, would improve predictions in less related populations (Pollak et al., 2012). However, relevance of marker density, size and relationship within and across training and selection population are still being discussed. Additionally, beef cattle population are less uniform compared to dairy cattle and both *Bos taurus* and *Bos indicus* are being used (Garrick, 2011). Better collation of genotype data, cross-comparison with large training populations (using higher density marker panels), possibly inclusion of females into the training data set and more emphasis on phenotyping might improve the potential for GS in beef cattle.

### IMPLEMENTATION OF GENOMIC SELECTION IN PIGS

In pigs, performance tests for offspring from selection candidates are completed in testing stations to reduce interactions through environmental factors. Animals are, in some countries such as Germany or Sweden, additionally tested on commercial farms to integrate variable environments into the selection process. Improvement of the genetic merit of pig populations is mainly based on sire selection and AI is commonly used. Most breeding schemes use crossbreeding between paternal production-oriented breeds and maternal reproduction-oriented breeds, and selection is mainly performed in purebred nucleus herds (Visscher et al., 2000). Sires lines are selected for improved carcass and meat traits, aiming to predict the performance of crossbred offspring.

Genomic selection in pig breeding is a potential tool to improve maternal traits as traditional breeding programs focus on performance traits in the sire lines. A simulation study has shown that GS has the potential to increase genetic gain for maternal traits in pigs (Lillehammer et al., 2011). A simulation study for the implementation of GS in male lines also concluded that the phenotypic selection can be improved while being (based on the selected genotyping strategy) economically efficient (Tribout et al., 2012).

Genomic selection might also assist to increase genetic gain in developing countries in which indigenous and exotic lines could be used for repeated backcrossing (Akanno et al., 2013). The use of genomic information will, also in such schemes, assist to control inbreeding and avoid a possible loss of variation.

Lower genetic correlations between traits in cross- and purebred animals (Dekkers, 2007) are reasons that GS has not yet been used as a selection tool with the same reliability as in dairy cattle. Many production traits are measured in crossbred pigs, while selection is based on the purebred lines they derived from. The integration of QTL information into breeding decision might enable to predict non-additive effects. However, inclusion of data from crossbred animals is required to reach higher accuracy compared to phenotypic selection (Dekkers and Chakraborty, 2004). Biologically meaningful markers, such as causative genes, candidate genes within gene-pathways or markers in strong linkage disequilibrium (LD) with the causative mutation might overcome the poor prediction of crossbred performance using information from purebreds only. It has been suggested that the use of MAS might assist to increase accuracies and thus genetic gain as it overcomes the difficulties especially for traits which are genetically different in purebred and commercial crossbred pig populations, for example those influenced by the environment (Dekkers, 2007).

Economic motivations are relevant for a successful implementation of GS in pig breeding, the reduction of the generation interval is significantly less compared to dairy cattle and current traditional selection methods do work well, despite the high costs for progeny testing. In 2014, one large, world-wide running pig breeding company announced their use of GS in crossbred populations^2^. However no details of the prediction method and accuracies have been given.

### SUGGESTED BREEDING PROGRAMS IN CHICKEN

Chicken breeding programs are in a pyramid form and comparable to pig breeding (Dekkers, 2007). The larger number of offspring in chicken allows more than double the genetic improvement compared to cattle or pigs when using traditional breeding. The implementation of GS in chicken has been discussed as it would reduce the generation interval from 12 to 6 months. It has been stated that costs will be a major part of the decision making process in scenarios for which genotypes are added to the existing performance testing scheme. Changes to most of the current breeding programs have been suggested, before a cost-efficient implementation of GS can be realized. The main change is the reduction of the number of animals within the program while keeping the effective population size the same (Sitzenstock et al., 2013). *In silico* studies have also suggested the implementation of GS in broiler lines, for which genotyping strategies need to be chosen carefully to reduce costs but still provide the full information (Avendaño et al., 2010). Also a layer breeding program with 250 males and females (compared to 1000 males and 3000 females in traditional breeding) would still lead to reasonably good predictions (Avendaño et al., 2010). Using a similar scenario, with 293 males and 913 females across four generations in the training population did also illustrate the potential of GS (Wolc et al., 2010).

But theoretical studies could, until now, not provide a clear answer on how genomic breeding values will be predicted in cross-breeding schemes, which are also commonly used in chicken breeding. It is possible that predictions made in genetically relatively close purebred lines will give reliable estimates for improvements in crossbreds, if QTL are inherited with the same marker allelic variants. However, another explanation for differences between predictions from purebred in crossbred populations is the difference of environmental factors. In one of the scenarios suggested by Sitzenstock et al. (2013) crossbred hens were therefore chosen from the environment closest to the production system, since housing conditions in purebred lines are often highly standardized.

However, side-effects could be a problem in most scenarios tested so far: one study observed a decreased egg weight, possibly as the consequence of improved feed efficiency of laying hens (Sitzenstock et al., 2013). Those scenarios also suggest that breeding organizations need to decide if faster genetic improvement of the breeding population will be a strong enough argument for a genomic enhanced, but more costly, selection scheme (Sitzenstock et al., 2013), taking possible risk of unwanted effects. It is in general important to carefully define breeding goals and indices to minimize negative effects on other economically relevant traits.

### CONSTRAINS IN FISH

Aquaculture is a broad field and combining hundreds of species under one header might not reflect the real potential and importance of aquaculture for provision of food. Also the opportunities and breeding programs vary widely in aquaculture as do generation intervals. There are only a few reports on GS in aquaculture in general and among the most important farmed fish species, studies are currently only published for the Atlantic salmon (Sonesson and Meuwissen, 2009; Nielsen et al., 2011; Lillehammer et al., 2013). The transfer of genetic gain from nucleus breeder to multiplier to commercial farms is, due to the high reproduction capability in fish, high and fast. The structure of the Norwegian Atlantic salmon breeding scheme allows approximately 8–10% genetic gain per generation for some traits (Gjoen and Bentsen, 1997). However, inbreeding might be higher if fewer breeding animals are kept (Gjedrem, 2005).

Current family-based breeding schemes in aquaculture, using information from close relatives (e.g., sibs) to estimate breeding values include only approximately half of the genetic variation into the selection decision (Sonesson and Meuwissen, 2009). A simulation study testing a GS-based sib-testing breeding scheme generally reduced the total genetic gain. It was suggested that aquaculture breeding programs would need to be re-designed to accommodate GS as the high genotyping costs could only be covered if very high genetic gain can be achieved (Sonesson and Meuwissen, 2009). Such changes could for example include fewer families, reduced phenotypic evaluation or use of field data from commercial farms, and either higher costs or reduced genetic gain have to be considered as consequences (Sonesson and Meuwissen, 2009; Nielsen et al., 2011). Achieved genetic gain would be higher at increased costs due to genotyping compared to conventional BLUP based breeding (Sonesson and Meuwissen, 2009). Breeding programs could be optimized in a way that pre-selection based on easy measureable but economic relevant traits like growth, would reduce the number of selection candidates (Sonesson and Meuwissen, 2009). It has been shown that GS controls inbreeding more effectively while allowing for improved genetic gain in a sib-based compared to a traditional BLUP breeding scheme (Nielsen et al., 2011). Reasons for increased inbreeding in BLUP breeding schemes are the co-selection of sibs in sib designs due to truncation selection on EBVs (Sonesson and Meuwissen, 2009), greater reproductive capacity of males and females, and inability to use within-family variation (Nielsen et al., 2011).

A combination between traditional BLUP estimation, pre-selection of candidates and low-density genotyping arrays might be one possibility to overcome the difficulties of high costs without high loss of genetic gain (Lillehammer et al., 2013). Alternative strategies, such as phenotypic recording from double haploids, which are applicable to fish populations, have been simulated and suggested as additional test population to gain genetic information. It has been shown that a careful design of test versus selection population for example using double haploids has the potential for increased selection accuracy compared to traditional sib testing schemes especially for traits that are difficult to record such as disease resistance or filet quality (Nirea et al., 2012).

## SUMMARY AND CONCLUSION

We reviewed here the approaches of GS in different livestock species and identify restrictions based on current breeding schemes. This article might provide a basis for the critical reading of articles on GS and thus interpretation of reported results and accuracies. This in turn could contribute to a broader range of viewpoints in future articles. There are more complex aspects to the topic of GS and issues mentioned here, such as assignment of varying effect sizes to markers, modeling of gene-by-gene or genotype-by-environment interactions or use of imputed genotypes and/or phenotypes. Further detailed analysis will be needed to allow a comprehensive demonstration on how to solve them in breeding schemes and selection decisions.

The title of this review emphasizes the diversity of current breeding schemes, and the need to either implement GS into existing structures or change breeding plans to fit novel breeding tools as suggested by others in more detail (e.g., Henryon et al., 2014; Ibanez-Escriche et al., 2014; Van Eenennaam et al., 2014). We summarized that even in dairy cattle populations, for which GS is widely applied, different schemes for the implementation of GS are being offered, allowing a faster or more conservative selection strategy. One of the main issue is that genomic predictions need to be reliable over many generations and the long-term efficiency of GS has yet to be proven in most practical applications. Tools for GS are available and genetic marker information can be implemented into breeding programs, combined with information on phenotypes and pedigrees of larger groups of individuals via complex algorithms. However, more questions have arisen with increased knowledge of the theoretic background and options of methodologies and technologies.

Some breeding practices make use of non-additive effects such as heterosis in cross-breeding. Genetic markers are often restricted to the prediction of additive effects, more complex mechanisms which cannot be explained by the DNA sequence information will be ignored when using such information for a breeding decision. Discussion will continue on the relevance of marker density, population size and structure to allow reliable estimations of the QTL effects and possibly diminish the size of not-explained effects by using genetic markers. Other uncertainties in the prediction of individual productivity or phenotypic appearance can occur due to inbreeding depression. And finally, the combination of (non-homozygote) genetic information is generated at random and combinations of non-linked loci can only be predicted in terms of probabilities.

The extensive number of publications and studies on models and methods for GS in different species indicates the potential of this still relatively novel breeding tool. Nevertheless, further research collaborations have arisen from some of the challenges that were identified, including the need for denser marker sets, which can now be further augmented since full genome sequences of many bulls are being collated (Daetwyler et al., 2014). Further studies using more applied populations and relevant traits need to proof if the expectations can be fulfilled. Despite some of the concerns, such as possibly higher costs due to genotyping not met by economic gains, GS has a future, since it holds more advantages such as control of inbreeding and known heritable defects or functional mutations, which again do add secondary advantages for marketing and product branding. A linked review on GS in crop species in the same research topic, aims to further deepen the relevance for an extended community.

## AUTHOR CONTRIBUTIONS

EJ and D-JK discussed the different aspects of genomic Selection; EJ wrote the first draft of the paper, while the final manuscript was written in collaboration.

### Conflict of Interest Statement

The authors declare that the research was conducted in the absence of any commercial or financial relationships that could be construed as a potential conflict of interest.

## Footnotes

1 http://www.interbull.org [accessed January 31, 2013]

2 http://www.pic.com/cms/USA/796.html
